# An occupational health survey on health utility and occupational diseases in Chinese university staff to inform cost-utility analysis

**DOI:** 10.3389/fpubh.2022.1022344

**Published:** 2023-01-10

**Authors:** Xiaoyan Liu, Huijun Zhou, Jie Wei, Minghui Li, Guofen Luo, Nasheen Naidoo, Guang Zhang, Ye Bi, Mengmeng Gao

**Affiliations:** ^1^Business School, University of Shanghai for Science and Technology, Shanghai, China; ^2^Department of Medical Affairs, The First People's Hospital of Tai'an, Taian, Shandong, China; ^3^Department of TCM Manipulative Orthopedics, PLA Air Force Medical Center, Beijing, China; ^4^Department of Clinical Pharmacy and Translational Science, University of Tennessee Health Science Center, Memphis, TN, United States; ^5^Department of Pathology, Stellenbosch University, Cape Town, South Africa; ^6^I.baby Preconception Care, Shanghai, China

**Keywords:** health utility, health related quality of life, EQ-5D-5L = EuroQol 5-dimensions 5-level, real-world evidence (RWE), university staff, occupational health

## Abstract

**Background:**

The occupational health of university staff bears great social and economic value for which health utility is an indivisible aspect. Utility is also the primary data for the cost-utility analysis of occupational health programs. Health utility and occupational diseases have not been reported for the university staff in China. In the light of “Healthy China,” we conducted this study aiming to (1) estimate the health utility of university staff to inform cost-utility analysis and (2) screen and identify potential occupational diseases for this occupation and examine their impacts on health.

**Methods:**

An occupational health survey was conducted in a sample of working-age university staff. Participants were interviewed face-to-face using the WHO Health and Work Performance Questionnaire and the European Quality of Life 5 Dimensions (EQ-5D) instrument to measure health conditions and health utility, respectively. The univariate analysis included the *t*-test, chi-square test, and correlation techniques. Multivariate generalized linear models were applied to evaluate the significance of each health condition when controlling for other factors.

**Results:**

The sample (*n* = 154) had a mean age of 40.65 years and consisted of slightly more women (51.30%). Participants attained a mean (standard deviation) health utility of 0.945 (0.073). The most affected domain was anxiety/depression with 62 (40.26%) participants reporting problems, followed by pain/discomfort which captured 60 (37.66%) staff with problems. Thus, pain and psychologically related conditions were prevalent. Multivariate models identified two conditions that can significantly reduce the health utility. The psychological/emotional conditions were associated with a utility loss of −0.067 (95%CI: −0.089, −0.045). The pain in body parts other than the head, neck, and back reduced the utility by −0.034 (95%CI: −0.055, −0.014).

**Conclusion:**

Working-age staff in Chinese universities may have a lower health utility than the general population. Psychological conditions and musculoskeletal pain appear like occupational diseases. With the health utility data available, economic evaluation of cost-utility should follow up to facilitate the implementation of cost-effective programs.

## Introduction

Occupational health is not only a public health issue but also an economic issue *per se*. Work-related health problems have caused tremendous loss to individuals, families, organizations, and society (1, 2). University staff constitute a unique occupational group in that they are highly skilled and highly educated, and their jobs demand intensive physical and mental efforts. Additionally, academics in the higher education sector are tasked with cultivating competent graduates and progressing science and culture to better society. Thus, the ill-health of this population has extensive social, economic, and cultural implications.

Although the occupational health of university staff bears great value, it has been largely ignored in China. This is most likely attributed to their work environment which is free of noise, dust, or heat. Occupational hazards are not easy to identify and occupational diseases are hard to define in a university setting. With that being said, studies have reported that mental disorders and musculoskeletal problems were common in this profession (3). In parallel with these reports, burnout, low job satisfaction, and occupational stress were frequently reported (4–6). Occupational factors such as work overload, sedentary work style, and long hours of lecturing have been blamed for the suboptimal health and health-related quality of life (HRQoL) (7–10). Therefore, university staff, just like workers in mining, construction, and manufacturing, maybe another health-disadvantaged population.

Occupational health and safety (OHS) programs have been proven effective in promoting the health of the workforce, reducing sickness absence, and improving the productivity of the organization and the sustainability of the business (11). The limited resources prohibit employers to invest in OHS fearing that it is not an economically wise investment. Economic evaluations in occupational health had the promise to address the issue (2). Nevertheless, the endeavors have been hampered by data deficiency, such as the health utility data of the employees for a cost-utility analysis (CUA) (2, 12).

CUA is a well-developed, full economic evaluation approach to inform the worthiness of health measures. CUA studies have been used to inform policies and programs in occupational health (13–15). Health utility derived from HRQoL is the primary input for a CUA. Moreover, HRQoL or health utility is in itself an important topic in occupational health research (16). Studies in China have suggested that HRQoL remains a concern in the population of university staff (4–7, 17). Unfortunately, none of these studies has reported health utility to support the CUA research in occupational health.

Recognizing this gap, we surveyed a school of a public university using the WHO Health and Work Performance Questionnaire (HPQ) and European Quality of Life 5 Dimensions 5 level (EQ-5D-5L) HRQoL instrument. The study set two objectives: (1) To estimate the health utility of university staff to inform occupational CUAs; and (2) to screen and identify potential occupational diseases for this occupation and examine their impacts on health utility. All in all, the study aspires to facilitate economic research in occupational health and inform the design and implementation of cost-effective OHS programs for university staff.

## Methods and materials

### Study setting and data collection

This was an occupational health survey conducted at the Business School, the University of Shanghai for Science and Technology, which is a public university in China. The eligibility criteria included: (1) official employee of the school; (2) not hospitalized or immediately after hospital discharge; (3) not handicapped or disabled; (4) able to carry out day-to-day work normally; and (5) able to give personal consent. The study was approved by the IRB committee of the Air Force Medical Center in Beijing.

The sampling frame consisted of 216 employees including both academic and administrative staff. The staff were personally invited through email, WeChat, or face-to-face contact. The recruitment advertisement was also announced at school-level meetings and internal WeChat workgroups. During the study period from 1 November 2020 to 15 January 2021, 155 people participated in the study, giving rise to a response rate of 78%. Seventeen persons were unavailable for reasons such as studying overseas, long-term sick or parental leave, hospitalization, resignation, or near-retirement. Another 35 staff were out of contact. Only nine personnel officially rejected the invitation. All participants were briefed about the study and then signed the informed consent form.

Data were collected through face-to-face interviews. Participants completed the questionnaires in the presence of the interviewer. The interviewer team consisted of one postgraduate and four undergraduate students who had attended three training sections each lasting 2 h. This pre-interview training aimed to ensure equivalent task understanding, procedures, and interactions with subjects. Interviewers were trained to give briefings and answer questions only, and not to promote a “right” or “wrong” answer as a measure to minimize the social desirability effect.

### Health utility measurement

We used the Chinese version of the EQ-5D-5L instrument to measure health utility (18). The EQ-5D is a preference-based HRQoL instrument asking participants to rate their present-day health. Compared to the old version of the EQ-5D-3L instrument, the EQ-5D-5L instrument is more favorable for HRQoL measurement due to its greater discriminatory power and lower ceiling effect (19, 20). Its validity and reliability have been validated in various Chinese populations (19, 21, 22).

The EQ-5D instrument has two parts. The first part is the descriptive system which classifies 3,125 health states into five health domains, that is, mobility (MO), self-care (SC), usual activities (UA), pain/discomfort (PD), and anxiety/depression (AD), each with five ordinal severity levels (no problems, slight problems, moderate problems, severe problems, and extreme problems/unable to). A respondent rated his/her health subjectively against the most appropriate statement in each of the five dimensions based on their health on the day of the interview. The scores of the five dimensions were used to calculate health utility according to the Chinese-specific value set (23). Health utility by definition has a range of 0 (death) to 1 (full health). The second part is called the visual analog scale (VAS), a 10-cm vertical bar anchored at 0 (worst imaginable health) and 100 (best imaginable health). The VAS indicated the overall health status rated by the respondents themselves.

### Occupation-related health conditions

Occupation-related health conditions were investigated using the WHO HPQ (24). In completing the HPQ, subjects need to report a wide range of health conditions of diseases and symptoms. HPQ is a self-report instrument designed to estimate the workplace costs of health problems. Its validity in studying chronic diseases relating to the workplace has been proven satisfactory (25). The questionnaire also collected information about other health determinants such as demographic (age, gender, height, and weight), lifestyle or behavioral (smoking and drinking), and socioeconomic (education, marital status, and number of children) factors (26).

### Statistical analysis

One participant was removed due to missing data. The final sample size for analyses was 154 participants. Categorical variables were described as counts and proportions. The prevalence of health conditions was estimated as the proportion of the sample presenting with certain health conditions. Continuous variables were presented as the mean and standard deviation (S.D.). The skewness and kurtosis of continuous variables were also explored to choose the appropriate models for multivariate analysis.

BMI was categorized into three groups—underweight/normal weight, overweight, and obese using a gender-specific standard for Chinese populations (27, 28), that is, 22.5 and 25.9 kg/m^2^ as cutoff points to define overweight and obesity in men, and 22.8 and 26.6 kg/m^2^ in women. Marital status was dichotomized into alone (single, divorced, or widowed) and married. A total of 40 health conditions were reported. This was too many to be treated as independent variables in the multivariate analysis for the sake of statistical power given the sample size. To carry out valid multivariate analyses, the six most prevalent conditions (≥20%) were treated as independent variables. These conditions were back/neck pain, pain in other areas, fatigue, high blood cholesterol, headache, and sleeping problems. The remaining 34 conditions were combined into seven groups by anatomical and/or physiological system (allergy, high blood pressure/cardiovascular, digestive system, respiratory system, musculoskeletal, psychological/emotional conditions, and others).

The univariate analysis involved correlation analysis between health utility and individual continuous variables, and *t*-test and ANOVA comparing the utility of different risk levels of categorical variables. A generalized linear model (GLM) was chosen to perform multivariate analyses as the dependent variable, EQ-5D utility, followed by a negatively skewed distribution (skewness = −1.89) to which GLMs were immune. The multivariate analysis evaluated the associations of health utility with sample characteristics. To remove the effect of collinearity and to produce consistent coefficients, the GLMs were set to use robust methods for parameter estimation and profile likelihood methods for confidence intervals. Unlike ordinary linear regression which models the raw data of the dependent variable, GLMs model the means of the dependent variable. Thus, in our study, the parameter coefficients of the GLMs represented the mean utility change associated with the specific variables.

All health conditions, except the category of other conditions, were evaluated simultaneously in one model together with demographic and socioeconomic variables. Doing so was to minimize collinearity because all the participants with other health conditions already had one or more classified diseases/symptoms. The analysis took *p* < 0.05 as being statistically significant. SPSS version 19 (SPSS Inc) was used for the analysis.

## Results

The characteristics of the sample are presented in Table 1. Our sample belongs to the working-age population and therefore had a mean age of 40.65 years with an upper age limit of the retirement age of 60 years. The mean BMI was 23.49 kg/m^2^ indicating that the enrolled staff were generally overweight for men and women alike (27). Only seven (4.55%) respondents had a Bachelor's degree. The majority (83.77%) were married and living with their families. For those raising children, the majority had one child only which was consistent with the One-Child policy in China. Up to 74% of the sample never smoked whereas half had the experience of drinking alcoholic beverages.

**Table 1:** Characteristics of the sample.

**Variables**	**Mean (S.D.)**	**Range (minimum, maximum)**	**Skewness/** **Kurtosis**
Age	40.65 (8.5)	(23, 60)	0.45/−0.47
BMI (kg/m^2^)	23.49 (4.36)	(16.14, 49.31)	2.24/9.61
	**Category**	***n***	**Percentage (%)**
Gender	Female	79	51.30
	Male	75	48.70
BMI	Under weight	8	5.19
	Normal weight	65	42.21
	Overweight	49	31.82
	Obesity	32	20.78
Highest academic degree	Bachelor's degree	7	4.55
	Master's degree or higher	147	95.45
Marital status	Living alone	25	16.23
	Married	129	83.77
Number of child	No child	39	25.32
	One child	88	57.14
	Two children	27	17.53
Smoking	Never	113	73.38
	Past	29	18.83
	Current	12	7.79
Drinking	No	77	50.00
	Yes	77	50.00

The overall prevalence of health conditions was 81.17% covering 40 diseases or symptoms. Half participants had back and/or neck pain reflecting a sedentary work style. The conditions inflicting eight (5%) or more subjects were summarized in Table 2. There were six conditions with a minimum prevalence of 20% (in descending order: chronic back and/or neck pain, pain in other body parts, chronic fatigue or low energy, high blood cholesterol, headache, and sleeping problems).

**Table 2:** The health conditions affecting 5% or more of the sample.

**Symptoms/diseases**	***n***	**Percentage (%)**
Back and/or neck pain	76	49.35
Pain in other body parts	55	35.71
Fatigue or low energy	47	30.52
High blood cholesterol	46	29.87
Headache	35	22.73
Sleep problems	31	20.13
Frequent diarrhea/constipation	29	18.83
Chronic heartburn/GERD	28	18.18
High blood pressure/hypertension	25	16.23
Anxiety disorder	24	15.58
Frequent nausea/gas/indigestion	23	14.94
Seasonal allergies/hay fever	22	14.29
Arthritis	22	14.29
Other emotional conditions	22	14.29
Stomach/intestine ulcer	16	10.39
Depression	14	9.09
Osteoporosis	13	8.44
Severe headaches	11	7.14
Chronic bronchitis	10	6.49

**Figure 1:**
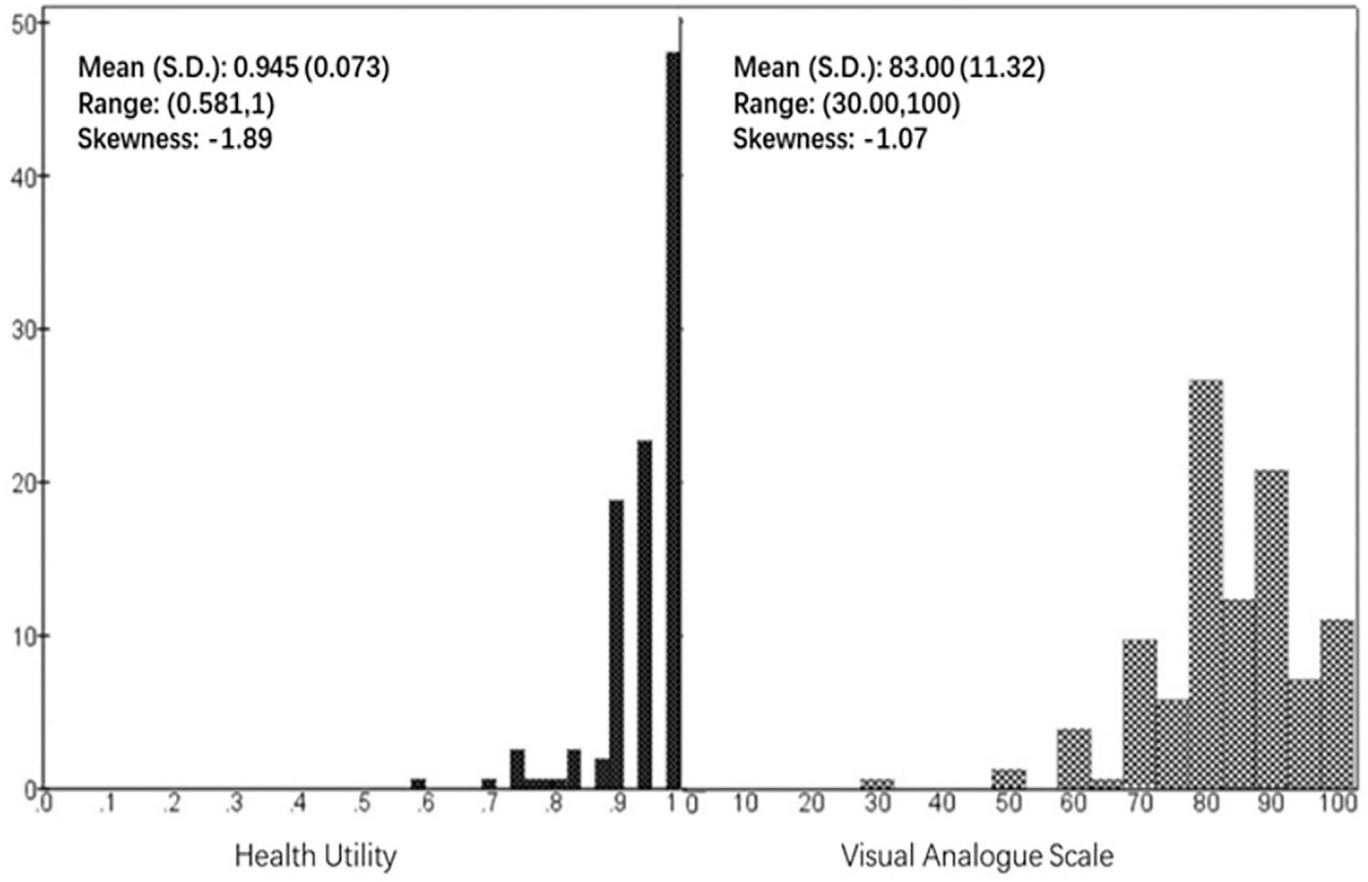
Distribution of health utility and visual analog scale.

Despite the symptoms and diseases reported, the overall mean (SD) utility was 0.945 (0.073), with only 0.055 (5.5%) utility loss compared to the full health of 1 (Figure 1). The mean VAS score was 83.00 (11.32), which was 17% lower than 100, the score indicating the best imaginable health. Both health indices followed negative distribution and were highly correlated (*r* = 0.595, *p* < 0.001).

**Figure 2:**
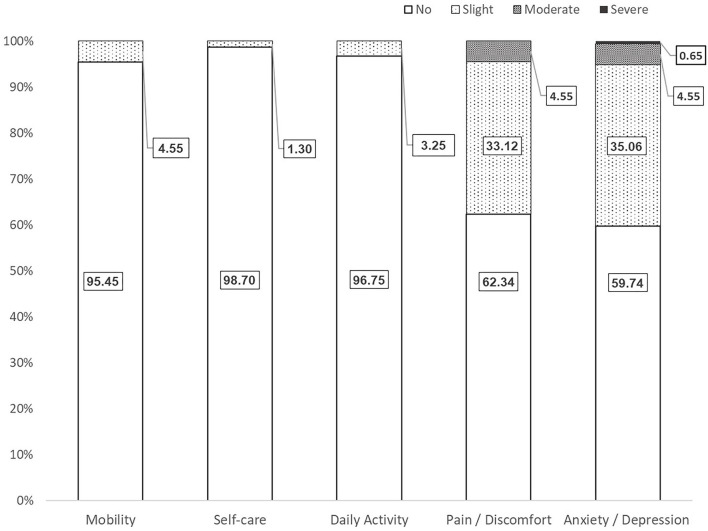
Health-related quality of life profile in EQ-5D domains. Numbers are percentages.

The health profile of the sample was reflected by the five EQ-5D domains in Figure 2. There were 74 (48.1%) participants who did not report any problems in the five domains, that is, they rated themselves in full health. This proportion also indicated the ceiling effect of the EQ-5D-5L instrument from the measurement perspective. The most affected domain was AD. Overall, 40.26% of participants reported anxiety or depression problems, one subject had a severe problem, seven subjects had moderate problems, and 54 subjects had slight problems. The second most affected domain was PD, in which 37.66% of the sample reported problems including seven subjects with moderate severity. On the other hand, very few participants reported problems in the MO, SC, and UA domains. None of these domains captured a case with problems more severe than slight problems.

As shown in Table 3, age and number of health conditions were negatively correlated with utility. Univariate analysis did not find significant comparisons of health utility for demographic and socioeconomic factors. For health conditions, all conditions except respiratory system conditions were associated with significant utility loss. Those subjects living with any condition reported lower utility than those without. The largest utility loss was related to psychological/emotional conditions. The subjects presenting with psychological/emotional conditions had a mean utility of 0.88, which was 0.09 lower than those without.

**Table 3:** Comparisons of health utility among different levels of each factor.

**Factors**		***n***	***r* ^a^**	**95% C.I**.	***P***
Age (years)		154	−0.16	(−0.32, −0.002)	0.045
Number of conditions		154	−0.56	(−0.68, −0.43)	< 0.001
	**Category**	***N***	**Mean**	**95% C.I**.	***P***
Gender	Female	79	0.94	(0.93, 0.96)	
	Male	75	0.95	(0.93, 0.96)	0.72
Education	Bachelor's degree	7	0.98	(0.96, 1.01)	
	Master's degree or above	147	0.94	(0.93, 0.96)	0.157
BMI (kg/m^2^)	Normal/under weight	73	0.95	(0.93, 0.97)	
	Overweight	49	0.95	(0.94, 0.97)	
	Obesity	32	0.92	(0.89, 0.96)	0.176
Marriage status	Remain alone	25	0.94	(0.91, 0.97)	
	Married	129	0.95	(0.93, 0.96)	0.534
Number of Child	0	39	0.94	(0.91, 0.96)	
	1	88	0.94	(0.93, 0.96)	
	2	27	0.96	(0.94, 0.98)	0.364
Smoking	Never smoked	113	0.94	(0.93, 0.96)	
	Past smoker	29	0.95	(0.93, 0.98)	
	Current smoker	12	0.96	(0.91, 1.01)	0.611
Drinking	No drinking	77	0.94	(0.93, 0.96)	
	Light drinking	77	0.95	(0.93, 0.96)	0.767
Allergy	No	132	0.95	(0.94, 0.96)	
	Yes	22	0.92	(0.87, 0.96)	0.036
High cholesterol	No	112	0.96	(0.94, 0.97)	
	Yes	42	0.92	(0.89, 0.95)	0.003
Back/neck pain	No	78	0.97	(0.96, 0.98)	
	Yes	76	0.92	(0.9, 0.94)	< 0.001
Insomnia	No	123	0.95	(0.94, 0.97)	
	Yes	31	0.91	(0.88, 0.94)	0.004
Fatigue	No	107	0.96	(0.95, 0.97)	
	Yes	47	0.91	(0.88, 0.93)	< 0.001
Pain in other body parts	No	99	0.97	(0.96, 0.98)	
	Yes	55	0.90	(0.88, 0.93)	< 0.001
Headache	No	116	0.96	(0.94, 0.97)	
	Moderate	27	0.93	(0.9, 0.95)	
	Severe	11	0.88	(0.79, 0.98)	0.002
Cardiovascular condition	No	127	0.95	(0.94, 0.96)	
	Yes	27	0.92	(0.88, 0.96)	0.032
Digestive system condition	No	95	0.96	(0.95, 0.97)	
	Yes	59	0.92	(0.89, 0.94)	< 0.001
Respiratory system condition	No	140	0.94	(0.93, 0.96)	
	Yes	14	0.96	(0.92, 0.99)	0.530
Muscular-skeleton condition	No	124	0.95	(0.94, 0.97)	
	Yes	30	0.91	(0.87, 0.95)	0.003
Psychological/emotional condition	No	116	0.97	(0.96, 0.98)	
	Yes	38	0.88	(0.85, 0.91)	< 0.001
Other health condition	No	140	0.95	(0.94, 0.96)	
	Yes	14	0.91	(0.85, 0.96)	0.036

^a^Pearson correlation coefficient.

95% C.I., 95% confidence interval.

In the GLMs evaluating multiple health conditions (Table 4), psychological/emotional conditions were associated with the biggest mean utility loss of −0.063, followed by the education level of a Master's degree or higher and pain in other body parts, which were associated with a utility loss of −0.048 and −0.036, respectively. Notably male gender, marriage, smoking, or having children were associated with utility gain. However, these factors did not achieve statistical significance in predicting health utility.

**Table 4:** Multivariate GLM simultaneously evaluating associations of individual factors with health utility.

**Risk factors**	**Mean utility loss^a^**	**95% C.I**.	***P***
Age	−0.001	(−0.002, 0)	0.136
Male vs. female	0.003	(−0.017, 0.023)	0.789
Obesity vs. normal weight	−0.019	(−0.049, 0.011)	0.209
Overweight vs. normal weight	−0.005	(−0.02, 0.011)	0.578
Minimum Master's degree vs. Bachelor's degree	−0.048	(−0.082, −0.014)	0.006
Married vs. living alone	0.021	(−0.006, 0.049)	0.133
Two children vs. no child	0.018	(−0.012, 0.048)	0.233
One child vs. no child	0.006	(−0.021, 0.032)	0.673
Current smoking vs. no smoking	0.011	(−0.029, 0.051)	0.589
Past smoking vs. no smoking	0.009	(−0.011, 0.029)	0.392
Drinking vs. no	−0.007	(−0.025, 0.011)	0.447
Allergy vs. no	−0.020	(−0.059, 0.018)	0.299
High cholesterol vs. no	−0.016	(−0.036, 0.004)	0.120
Pain with back/neck vs. no	−0.005	(−0.03, 0.020)	0.679
Insomnia vs. no	−0.006	(−0.038, 0.027)	0.732
Fatigue vs. no	−0.015	(−0.035, 0.005)	0.135
Pain with other body parts vs. no	−0.036	(−0.061, −0.011)	0.004
Headache severe vs. no headache	0.002	(−0.060, 0.064)	0.949
Headache moderate vs. no headache	0.004	(−0.021, 0.029)	0.746
High blood pressure/cardiovascular condition vs. no	−0.005	(−0.030, 0.019)	0.677
Digestive system condition vs. no	−0.008	(−0.027, 0.011)	0.425
Respiratory system condition vs. no	0.030	(−0.007, 0.069)	0.061
Musculoskeletal condition vs. no	−0.010	(−0.042, 0.021)	0.517
Psychological/emotional condition vs. no	−0.063	(−0.090, −0.037)	< 0.001

^a^Negative sign indicates mean utility loss associated with the factor or vice versa.

95% C.I., 95% confidence interval.

## Discussion

Focusing on the HRQoL aspect of occupational health, our study estimated that the mean health utility was 0.945 for the university staff, while the subjective HRQoL was conservative with a mean VAS score of 83. The utility was closely linked to the AD and PD domains in which 40.26 and 37.66% of participants reported problems, respectively. The most prevalent diseases or symptoms in this occupation seemed to be broadly related to pain (back, neck, head, and other body parts) and mental problems (sleep problems, fatigue, and anxiety disorder). Psychological/emotional conditions, Master's degree, and pain in body parts were significant factors contributing mean utility loss of −0.067, −0.048, and −0.034, respectively. To the best of our knowledge, this is the first study specifically dedicated to the health utility of university staff in China.

Economic and social benefits are important considerations of employers in providing OHS services to their employees. Anyway, resources are limited and deserve cost-effective use. Following that, economic evaluations on OHS policies are common research practices in advanced countries (2, 12, 13, 29). Data availability always emerged as a barrier for such studies, which can only be worse in developing countries such as China. To conduct CUA in an occupational health setting, the utility data is harder to obtain than the cost data, as the latter is more direct for collection. Although health utility has been measured nationally with the EQ-5D-3L in China (National Health Services Survey, NHSS) once every 5 years since 2003 (30), the data are supposedly limited to inform the HRQoL of a specific occupational group. Additionally, the EQ-5D-3L tends to overestimate health utility for a generally healthy population (31). Compared to the EQ-5D-5L, EQ-5D-3L is less powered to discriminate between different health statuses and more affected by the ceiling effect (19, 20). Therefore, the health utility estimated by this study makes valid evidence for a CUA targeting at occupational health of university staff.

Unlike workers employed in hazardous occupations, university staff were unlikely to be considered a health-disadvantaged population. Our findings seemed to suggest otherwise. Compared to a survey attempting to create health utility norm in urban residents with EQ-5D-5L (32), their subjects (*n* = 965) of the same age as our sample achieved a mean utility of 0.961, which was higher than 0.945 for our sample. The difference does not reach statistical significance reflecting that the two study populations are generally considered healthy. Our participants reported more problems in all EQ-5D domains than the age-matched participants of the norm study. The differences in AD and PD were statistically significant (*P* = 0.001 and *P* = 0.016). Of our sample, 40.26 and 37.66% of subjects reported AD and PD problems, respectively, compared to 27.56 and 28.7% of age-matched subjects in that study (32). The health disadvantage of university staff was further supported by comparing our study with the 2013 NHSS enrolling 188,720 Chinese across mainland China (33). Our sample again had a significantly lower utility than the national sample (0.945 vs. 0.985, *P* = 0.011). Given that the national sample was heterogeneous with respect to health determinants, our sample was further compared to the subpopulation holding university degrees or higher, in that the education level is the distinctive characteristic of university staff and is closely relevant to other factors such as residence, employment, and health insurance. Compared to the education-matched cohort, the larger gaps in utility and VAS were revealed seeing that, for our sample of university staff, the utility shortage was 0.05 and the VAS shortage was 2.44 on average. Both differences were statistically significant (*P* < 0.001, *P* = 0.007).

Occupational diseases for university staff have not been defined in the National List of Occupational Diseases in China. According to our findings, pain and mental problems are popular medical complaints. Different types of pain all had relatively high prevalence (Table 2). Psychological and emotionally relevant problems such as fatigue or low energy, sleep problems, anxiety disorder, other emotional problems, and depression were also common or had a higher prevalence than the national figures (34). As for HRQoL, our participants were 7.68 times more likely to report PD problems and 16.10 times more likely to report AD problems than the education-matched NHSS sample (33). Even compared to other NHSS cohorts with similar socioeconomic characteristics of working age, employment status, or residence, the likelihood of PD and AD in our sample was three and eight times higher (33). Consistent with our findings, previous Chinese studies also reported poor mental health in university lecturers (3, 6, 35). Neck or back pain is popular among university employees in China and other countries and has caused considerable productivity loss due to sickness (8, 36–38). Our results are also in line with international studies showing that problems with mental health and the musculoskeletal system are the two major work-related medical conditions causing high costs to employers (39–41). Putting all evidence together, pain and mental disorders seem to be qualified as occupational diseases for the profession of university staff and are officially listed as so. A CUA study taking full account of cost and utility would produce convincing evidence for this purpose.

There were 63.64% of participants reporting physiological pain while only 37.68% reporting problems in the PD domain. Conversely, 24.68% of participants reporting psychological or mental conditions while 40.26% reporting problems in the AD domain. The health profile from a clinical perspective appears quite different from an HRQoL perspective. This disparity is most likely culture-related (42). The Chinese are not willing to admit their mental problems for fear of discrimination. The social stigma surrounding mental disease is stronger in China than in the West (43, 44). However, it is the opposite with respect to pain. Being traditionally viewed as diligent or industrious, the Chinese have a high tolerance for pain for fear of appearing lazy or irresponsible (45). This finding has highlighted the need to include the HRQoL in the basket of occupational health indicators to have a better understanding of the relationship between personnel's health and the organization's productivity (5, 7, 16).

There were several advantages to our study. The ceiling effect of the EQ-5D-5L in measuring our sample was 48% only, much lower than the 84.2% in the NHSS and similar studies in Asia, the USA, and Europe (46–50). The ceiling effect limits an HRQoL instrument to measure suboptimal health when subjects are generally healthy resulting in overestimated utility. A modest ceiling effect secures the reliability and validity of our findings of health utility. Another advantage is that we employed a GLM for multivariate analysis, which is superior to more commonly used linear regression models and produces more stable estimates for the study population.

Some limitations to our study are notable. The sample size is small which may affect its representativeness of the population of university staff in China. However, university staff are homogenous in terms of age, education level, job duty, and socioeconomic characteristics. This means that, statistically, a small sample may have good representativeness of the study population (51). The idea based on our findings that university staff have worse health status than the general population might be related to the choice of HRQoL instrument or measurement bias. Our study used the EQ-5D-5L instrument to measure HRQoL, whereas the NHSS studies used the EQ-5D-3L instrument (30, 33), which, due to its lower discretionary power, systematically generated higher health utility than the EQ-5D-5L instrument (19). This implies that the lower health utility in our sample might be spurious and introduced by the EQ-5D-5L. On the reverse side, compared with the more recent study that used the EQ-5D-5L (32), university staff again had lower health utility and significantly more problems in AD and PD. If the measurement bias ever existed, it would be less likely to bias our findings to a worrying extent. Finally, this study did not evaluate occupational factors, for example, working hours or workload, in terms of their impacts on the HRQoL. This might weaken the power of study findings as an occupational survey.

## Conclusion

Staff working in Chinese universities may have lower HRQoL and health utility than the general population. This indicates that occupational health in the higher education sector remains an indispensable health issue. Psychological conditions and musculoskeletal pain appear like occupational diseases specifically in the population of working-age university staff. The utility loss was mainly manifested with impairment in the AD and PD domains. With the health utility data available, economic evaluations such as CUA should follow-up on time to facilitate the implementation of cost-effective OHS measures.

## Data availability statement

The raw data supporting the conclusions of this article will be made available by the authors, without undue reservation.

## Ethics statement

The studies involving human participants were reviewed and approved by the IRB Committee of the Air Force Medical Center in Beijing. The patients/participants provided their written informed consent to participate in this study.

## Author contributions

XL conceptualized and designed the study. HZ conceptualized the study, analyzed the data, and wrote the original draft. JW and ML managed the project and interpreted the results. GL and NN interpreted the results and offered important ideas for discussion. GZ did the preliminary statistical and advised on methodology. MG and YB collected and compiled the data. All authors made major revisions to the draft and approved the manuscript for final submission.
